# Cutaneous nontuberculous mycobacterial infection after intra-articular corticosteroid injection in a patient with psoriatic dactylitis

**DOI:** 10.1590/0037-8682-0055-2025

**Published:** 2025-06-27

**Authors:** Vanessa Ribeiro Ferreira, Mariana Santiago Bernardes, Rebeca de Oliveira Alves Melo Martins, Isabella Cristiny Mendes Alexandre, Carolina Souza de Oliveira, Thalyssa Figueiredo Magalhães, Daniela Vale Dias, Laísa Ezaguy de Hollanda, Guilherme Augusto Pivoto João, Marcelo Cordeiro-Santos, Luciana Mendes dos Santos

**Affiliations:** 1 Hospital Universitário Getúlio Vargas, Departamento de Dermatologia, Manaus, AM, Brasil.; 2 Fundação de Medicina Tropical Dr. Heitor Vieira Dourado (FMT-HVD), Departamento de Infectologia, Manaus, AM, Brasil.; 3 Universidade Federal do Amazonas (UFAM), Departamento de Dermatologia, Manaus, AM, Brasil.

**Keywords:** Psoriatic dactylitis, Nontuberculous mycobacteria, Tumor necrosis factor blocker

## Abstract

The incidence of skin nontuberculous mycobacterial (NTM) infections has been rising in recent years, paralleling the increase in invasive procedures and immunosuppression. We present a case involving a patient undergoing treatment with a tumor necrosis factor blocker (Etanercept®) for psoriatic dactylitis, who showed clinical deterioration 30 days following intra-articular corticosteroid therapy. Liquid culture media identified *M. abscessus*, prompting the initiation of combination antibiotic therapy. To the best of our knowledge, this is the first reported case of an NTM superinfection in dactylitis, underscoring the importance of screening for these infections in immunosuppressed patients receiving localized injectable treatments.

## INTRODUCTION

Dactylitis is characterized by inflammation of an entire digit, causing uniform swelling. This swelling encompasses the soft tissues from the metacarpophalangeal to the interphalangeal joints, including the digital tuft, which gives the affected finger or toe a distinctive sausage-like appearance^1^ ^,^ ^2^ ^,^ ^3^. Dactylitis can be classified based on the tissues involved: bone only, both bone and soft tissue, or solely soft tissue. Additionally, it is categorized by its etiopathogenesis into noninflammatory, inflammatory infectious, and inflammatory noninfectious types^1^ ^,^ ^4^. 

Psoriasis, a chronic inflammatory immune-mediated disease, frequently causes inflammatory noninfectious dactylitis and is a key clinical feature of psoriatic arthritis (PsA). It affects 30-50% of PsA patients^2^. In such cases, diagnostic imaging, including magnetic resonance imaging (MRI) and ultrasonography, often reveals flexor tenosynovitis, marked soft tissue swelling, varying degrees of small joint synovitis, fluid accumulation in tendon sheaths, and an increased volar bone-to-skin distance primarily due to synovial thickening around the flexor tendons^1^ ^,^ ^4^. The management of PsA typically begins with a "step-up" approach, starting with non-steroidal anti-inflammatory drugs (NSAIDs) for pain relief and corticosteroids to reduce inflammation during the initial stages. Corticosteroids are usually given intramuscularly or intra-articularly, with the latter being preferred for persistent mono- or oligoarthritis. Treatment may escalate to disease-modifying antirheumatic drugs (DMARDs) and, if necessary, to biological agents that target tumor necrosis factor (TNF), IL-12/23, or IL-17 inhibitors^5^. While generally well tolerated, anti-TNF agents are associated with a heightened risk of granulomatous infections, including nontuberculous and tuberculous mycobacteria, particularly in endemic areas such as Brazil^6^.

Nontuberculous mycobacteria (NTM) constitute a diverse group of species that are distinct from the *Mycobacterium tuberculosis* complex and *Mycobacterium leprae,* first identified as human pathogens in the 1950s^7^ ^,^ ^8^. These ubiquitous, non-motile, acid-fast bacilli are found in significant reservoirs, including water, soil, dust, animals, vegetation, and hospital environments, notably on medical equipment such as surgical tools and endoscopes^7^ ^,^ ^8^ ^,^ ^9^. Infections may occur following surgery, cosmetic procedures, or traumatic injuries. While typically requiring a breach in the skin barrier, NTMs can also cause cutaneous infections through the systemic dissemination of the disease, particularly in immunocompromised patients^7^ ^,^ ^8^. NTMs exhibit moderate resistance to standard disinfectants, including chlorine, organomercurials, and alkaline glutaraldehydes. The increasing incidence of infections can be attributed to inadequate cleaning, disinfection, and sterilization practices, such as relying on these disinfectants for sterilizing solutions or using non-sterile water for cleaning surgical instruments^9^ ^,^ ^10^. Additionally, rapidly growing nontuberculous mycobacteria (RGNTM) such as *M. fortuitum*, *M. abscessus*, *M. chelonae*, and *M. conceptionense* have been implicated in healthcare-associated infection outbreaks linked to surgical procedures^9^ ^,^ ^10^.

We report the first documented case of a cutaneous nontuberculous mycobacterial infection, specifically *Mycobacterium **abscessus*** , in a patient with psoriatic dactylitis undergoing treatment with a tumor necrosis factor blocker following an intra-articular corticosteroid injection. A comprehensive literature review was conducted using the Cochrane Library, LILACS, SciELO, MEDLINE, PubMed, and PubMed Central databases, and no previous case reports with this correlation were found. 

## CASE REPORT

A 45-year-old male public servant from Manaus, Amazonas, diagnosed with psoriatic arthritis and undergoing treatment with a tumor necrosis factor blocker (Etanercept®), presented with a one-month history of swelling and pain in one finger. A rheumatological assessment revealed clinical and radiological signs of psoriatic tenosynovitis. The patient was prescribed systemic and intra-articular corticosteroid therapy, which resulted in partial improvement; however, clinical deterioration was observed 30 days later.

**FIGURE 1:**
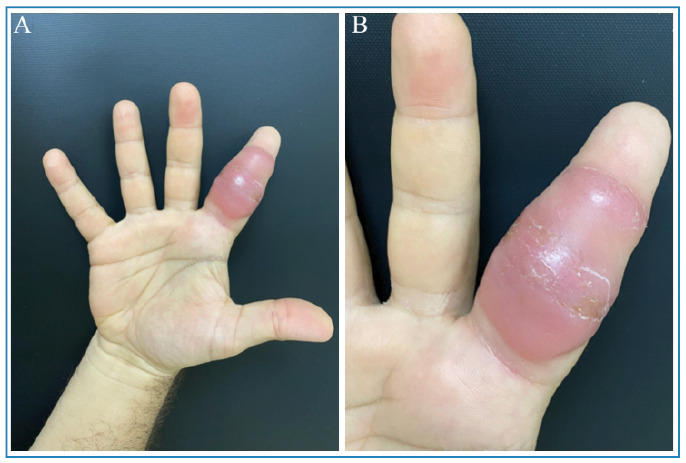
**A:** Erythema and swelling of the second digit of the right hand. **B:** Erythematous nodules with increased consistency.

**FIGURE 2:**
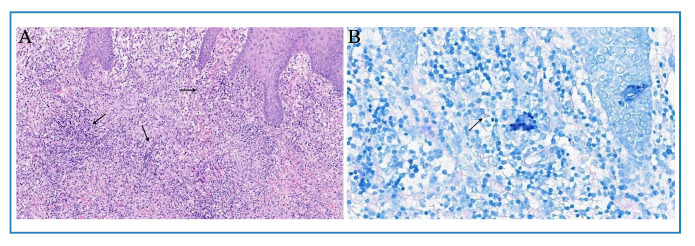
**A:** Dense lymphohistiocytic inflammatory infiltrate in the superficial and deep dermis (black arrows) (H&E 100x). **B:** Rare acid-fast bacilli visible through Ziehl-Neelsen staining (black arrow).

Upon examination, erythematous nodules with increased consistency were observed on the second digit of the right hand (Figure 1), accompanied by small nodules along the lymphatic pathway of the ipsilateral forearm. A skin biopsy was conducted, and histopathological analysis showed dense lymphohistiocytic inflammatory infiltrates in both the superficial and deep dermis (Figure 2A), with the presence of rare acid-fast bacilli (AFB) identified by Ziehl-Neelsen staining (Figure 2B).

Magnetic resonance imaging (MRI) of the second digit revealed marked tendinopathy and tenosynovitis of the flexors with significant fluid distension, pronounced diffuse synovial proliferation, and post-contrast enhancement, likely associated with psoriatic arthritis activity. Additionally, a discontinuity of the sheath in the volar region was observed, accompanied by extensive edema and post-contrast enhancement in the subcutaneous tissue of the volar area of the middle and proximal phalanges of the second digit. The MRI also showed a complete rupture of the flexor digitorum profundus with retraction and a substantial laceration of the flexor digitorum superficialis tendon at the metacarpophalangeal joint level, including partial fiber rupture and a small joint effusion in the proximal interphalangeal joint of the second digit. No signs of inflammatory or infectious involvement were noted in the bone structures. 

Diagnostic testing using GeneXpert® MTB/RIF for *Mycobacterium tuberculosis* and fungal evaluation via specific staining and culture yielded negative results. However, the liquid culture medium (BACTEC MGIT 960) confirmed the growth of *Mycobacterium abscessus*. Based on the identification of AFB, histopathological findings, and cultured mycobacteria, a diagnosis of skin and soft tissue infection caused by NTM was established. The patient began treatment with ciprofloxacin and clarithromycin.

**FIGURE 3:**
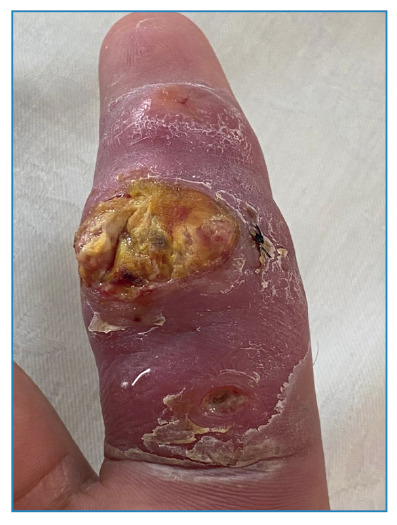
Ulcer with shallow borders and a vegetative, fibrinous base on the second digit of the right hand.

After one week of treatment, the patient developed an ulcer with shallow borders and a vegetative, fibrinous base (Figure 3). Following 45 days of modest response to treatment, ciprofloxacin was empirically replaced with moxifloxacin due to its broader intracellular penetration and more favorable in vitro activity against rapidly growing mycobacteria compared to other quinolones, despite the absence of antimicrobial susceptibility testing. This adjustment resulted in progressive clinical improvement.

## DISCUSSION

The incidence of skin NTM infections has risen notably in recent years, coinciding with an increase in invasive procedures and immunosuppression. Most of these cases are linked to invasive interventions, particularly aesthetic procedures^9^ ^,^ ^10^.

The use of cellular immunosuppressants, such as corticosteroids and tumor necrosis factor-alpha (TNF-α) inhibitors, significantly heightens the risk of infections. Notably, anti-TNF-α agents increase the risk of reactivating latent tuberculosis and NTM infections by impairing granuloma formation. TNF-α plays a crucial role in both the pathogenesis and maintenance of granulomas, aiding in the recruitment of monocytes and lymphocytes to the infection site. In established granulomas, the loss of TNF signaling undermines the structural integrity, boosts intracellular mycobacterial growth, and leads to the breakdown of granulomas, thereby releasing mycobacteria into the extracellular environment^6^.

Furthermore, invasive procedures and subcutaneous drug administration can compromise the skin’s protective barrier, elevating the risk of mycobacterial infections. These organisms, especially RGNTMs, are often responsible for outbreaks of skin and subcutaneous tissue mycobacterial infections and demonstrate resistance to standard disinfection protocols^9^ ^,^ ^11^ ^,^ ^12^.

Diagnosing NTM infections presents significant challenges. In this case, a skin biopsy was conducted, and samples were analyzed using bacilloscopy, cultured on specialized media, and subjected to histopathological examination. The diagnosis was suspected based on histopathological features consistent with suppurative granuloma^8^. The use of polymerase chain reaction (PCR) is recommended to enhance diagnostic accuracy, as it can detect NTM even when cultures return false-negative results^9^.

Treating NTM infections is also complex due to their limited susceptibility to conventional antimicrobials. Treatment typically starts with an empirically chosen combination of two or more antibiotics, tailored based on species identification-although this process is often complicated by the low sensitivity of diagnostic tests-and is maintained over prolonged periods^7^.

To our knowledge, this is the first reported case of an NTM superinfection associated with psoriatic dactylitis. It underscores the importance of prevention and screening for NTM infections in patients undergoing local injectable and anti-TNF therapies, especially in endemic regions such as Brazil.
